# Annotated Expressed Sequence Tags (ESTs) from pre-smolt Atlantic salmon (*Salmo salar*) in a searchable data resource

**DOI:** 10.1186/1471-2164-8-209

**Published:** 2007-07-02

**Authors:** Alexei A Adzhubei, Anna V Vlasova, Heidi Hagen-Larsen, Torgeir A Ruden, Jon K Laerdahl, Bjørn Høyheim

**Affiliations:** 1 The Biotechnology Centre of Oslo, University of Oslo, PO Box 1125 Blindern, NO-0317 Oslo, Norway; 2 Norwegian School of Veterinary Science, BasAM-Genetics, PO Box 8146 DEP, NO-0033 Oslo, Norway; 3 Engelhardt Institute of Molecular Biology, RAS, Moscow, Russia; 4 Centre for Molecular Biology and Neuroscience (CMBN) and Institute of Medical Microbiology, Rikshospitalet-Radiumhospitalet Medical Centre, NO-0027 Oslo, Norway

## Abstract

**Background:**

To identify as many different transcripts/genes in the Atlantic salmon genome as possible, it is crucial to acquire good cDNA libraries from different tissues and developmental stages, their relevant sequences (ESTs or full length sequences) and attempt to predict function. Such libraries allow identification of a large number of different transcripts and can provide valuable information on genes expressed in a particular tissue at a specific developmental stage. This data is important in constructing a microarray chip, identifying SNPs in coding regions, and for future identification of genes in the whole genome sequence. An important factor that determines the usefulness of generated data for biologists is efficient data access. Public searchable databases play a crucial role in providing such service.

**Description:**

Twenty-three Atlantic salmon cDNA libraries were constructed from 15 tissues, yielding nearly 155,000 clones. From these libraries 58,109 ESTs were generated, of which 57,212 were used for contig assembly. Following deletion of mitochondrial sequences 55,118 EST sequences were submitted to GenBank. In all, 20,019 unique sequences, consisting of 6,424 contigs and 13,595 singlets, were generated. The Norwegian Salmon Genome Project Database has been constructed and annotation performed by the annotation transfer approach. Annotation was successful for 50.3% (10,075) of the sequences and 6,113 sequences (30.5%) were annotated with Gene Ontology terms for molecular function, biological process and cellular component.

**Conclusion:**

We describe the construction of cDNA libraries from juvenile/pre-smolt Atlantic salmon (*Salmo salar*), EST sequencing, clustering, and annotation by assigning putative function to the transcripts. These sequences represents 97% of all sequences submitted to GenBank from the pre-smoltification stage. The data has been grouped into datasets according to its source and type of annotation. Various data query options are offered including searches on function assignments and Gene Ontology terms. Data delivery options include summaries for the datasets and their annotations, detailed self-explanatory annotations, and access to the original BLAST results and Gene Ontology annotation trees. Potential presence of a relatively high number of immune-related genes in the dataset was shown by annotation searches.

## Background

The role of aquaculture in world food industry has rapidly become more important in the last 20 years. Atlantic salmon is an important aquaculture species with an interesting biology. It spawns in fresh water and develops through several stages before migrating to the sea to feed, a dramatic change of habitat that requires physiological, morphological and behavioural changes. In addition the salmonids did undergo a duplication event 25–100 Myr ago [1] and show residual tetraploidity. Atlantic salmon also shows a very different recombination rate between females and males with females having normal recombination while males show a significantly reduced recombination rate [2,3]. As an important aquaculture species it is crucial to understand the biology of Atlantic salmon, its associated diseases, as well as environmental impact of salmon farming, e.g. effects of escaped fish on wild populations and health issues of interactions of farmed and wild fish. The fact that Atlantic salmon is a species that can yield both biologically revealing and practically important results makes it an important species for research, including genomic research.

The major goal of all farm animal genome projects is to identify the genetic mechanisms responsible for important and commercially interesting traits, such as disease resistance, growth, meat colour, fat deposition etc. in order to implement these results in the breeding and management programmes. Compared to most other farmed animals there is still a large stock of wild fish for most aquaculture species. This means that there is also a great need for managing the wild populations. To identify these genetic mechanisms one needs access to various tools such as a genetic and physical map, polymorphic markers (both microsatellites and SNPs), cDNA libraries, ESTs and full-length gene sequences, and preferably the whole genome sequence. In addition bioinformatics tools and databases are needed to extract biologically meaningful results from this large amount of data.

For Atlantic salmon some of these resources have been developed. They include genetic markers and maps [2-4], a BAC library [5] and the corresponding physical map [6]. Another important genomic resource are cDNA libraries from different tissues and developmental stages, and their relevant sequences (ESTs or full length sequences). Such libraries make it possible to identify a large number of different transcripts and can also give valuable insight into which genes are expressed in which tissue/library at a specific developmental stage. The existing cDNA resources include the EU funded SALGENE (Generation of a genetic body map for Atlantic Salmon) [7], the Canadian GRASP (Genomics Research on Atlantic Salmon Project) [8,9] and the Norwegian Salmon Genome Project (SGP) [10-12]. The three projects have used different strategies to construct and sequence the various libraries. While GRASP has developed mainly mixed tissue normalised libraries that has been sequenced from both the 5' and 3' ends, SALGENE and SGP developed tissue specific libraries and sequenced mainly from the 5' end. GRASP and SALGENE constructed most of their libraries from adult fish that has been adapted to the saline environment while SGP focused on the pre-smolt stage, i.e. on fish that were still living in fresh water.

There are currently in the order of 430,000 *Salmo Salar *EST sequences in GenBank, of these 55,118 EST sequences have been generated and submitted as part of the Salmon Genome Project. The DFCI (TIGR) annotated Atlantic salmon Gene Index (AsGI) [13] comprises 236,009 ESTs, 598 ETs (Expressed Transcripts) which after contig assembly yielded 29,544 TC (Tentative Consensus) sequences and 33,697 singlets. Such a resource is essential when constructing a microarray chip and identifying SNPs in coding regions, furthermore it provides a backbone for identifying genes in the future genomic sequence. Recently more than 2,500 putative SNPs have been identified in Atlantic Salmon of which a set of 65 were validated [14]. In addition, the EST sequences and the corresponding clones have been used to develop various microarray chips based on spotted cDNA clones [9].

This paper describes a combined biological and bioinformatics resource and database dedicated to EST sequences from tissue specific pre-smolt Atlantic salmon cDNA libraries. The resource combines a clone as well as a sequence repository and the results derived from data processing. In addition, this includes amplified stocks of libraries from each of the tissues used, which can be used for further studies.

## Construction and content

### Construction of cDNA Libraries

cDNA libraries were constructed from 15 tissues of pre-smolt Atlantic salmon. Each library was constructed using RNA from 5 individual fish to increase the probability of identifying SNPs in the resulting sequences. An exception was the library constructed from testis were we where only able to extract RNA from one individual. Library construction and enrichment of the cDNA libraries were performed as described earlier [12].

After construction the libraries were plated out on agar plates, grown overnight and subsequently picked and re-grown overnight in 96 well plates. On the next day the colonies were transferred to 384 plates in duplicate and stored as glycerol stocks at -80°C. Nearly 155,000 clones have been picked from the various libraries (Table 1). Two of the libraries (gills and intestine) were pre-screened to separate abundant clones from rare ones [12]. For all other libraries no pre-screening was performed in order to identify as many SNPs as possible. One exception was white muscle were we, after the initial sequencing of the first 1152 sequences had to make a pre-screening to reduce the amount of three genes that dominated the transcripts from this tissue (glyceraldehyde-3-phosphate dehydrogenase, creatine, aldolase). This was done by hybridising three clones, HM4_0714, HM4_0584 and HM4_0679 representing each of the three genes to filters containing the complete set of white muscle genes. After hybridisation, the non-hybridising clones were picked and re-arrayed into new 384 well plates. In addition, 4 SSH libraries were constructed after intra-peritoneal injection of infectious salmon anaemia virus (ISAV) [15].

**Table 1:** cDNA pre-smolt libraries used for contig assembly for the SGP dataset.

Library name	Number of sequences		
	Pre-processed (failed)	Submitted to GenBank	Selected for clustering

Brain	3991 (781)	3847	3991
Brain II	2263 (400)	1882	2263
Eye	5052 (1060)	4870	5052
Gills	3075 (762)	2964	3075
Gills II	1113 (164)	1053	1113
Head kidney	4358 (609)	4210	4358
Heart	1999 (288)	1732	1999
Heart II	1211 (405)	1141	1211
Intestine	2865 (708)	2737	2865
Kidney	1528 (1122)	1433	1528
Liver	2383 (292)	2312	2383
Ovaries	4142 (826)	4113	4142
Red muscle	661 (96)	629	661
SSH Gills down-regulated	446 (28)	262	269
SSH Gills up-regulated	415 (59)	247	248
SSH Intestine down-regulated	222 (65)	218	222
SSH Intestine up-regulated	252 (30)	247	252
Skin	1084 (64)	1010	1084
Spleen	4624 (517)	4576	4624
Swimbladder	2245 (419)	2182	2245
Testes	4910 (732)	4854	4910
White muscle	7316 (653)	6656	6763
White muscle II	1954 (294)	1943	1954

Total	58109 (10374)	55118	57212

Full statistics on the SGP and other libraries are available at SGP data resource > Data and results > cDNA libraries > SGP libraries list, or individual libraries.

### DNA sequencing

Sequencing was done from the 5'-end using T3 as sequencing primer. 5'-sequencing was chosen in order to assign functional annotation to as many transcripts as possible. The sequencing reactions were performed using the ABI PRISM^® ^BigDye™ Terminators Cycle Sequencing Kit (Applied Biosystems), and run on the ABI 377 (Applied Biosystems) (Gills and Intestine [12]) or using the MegaBACE DYEnamic ET dye terminator kit on the MegaBACE 1000 (Amersham Pharmacia) (all other libraries/sequences). The sequencing was performed according to the manufacturers protocols. When using the MegaBACE DYEnamic ET dye terminator kit, DNA was amplified using TempliPhi from Amersham. One microlitre of stock culture was transferred to a new 384 well-plate and 10 μl of denaturation buffer was added. The sample was denatured at 95°C for 3 minutes, transferred to ice and 10 μl of premix was added before the samples were placed at 30°C for 12 hours. From this reaction 0.4 μl was mixed with 4 μl of sequencing mix, 4.8 μl of water and 0.5 μl of T3 primer (5 μM). The sequencing reaction was done by 40 cycles at 95°C for 20 sec., 50°C for 15 sec. and 60°C for 2 minutes. Before the samples were loaded on the MegaBACE the excess dye terminators were removed using a Dye Terminator Removal Kit from ABgene.

### Database, sequence processing and clustering

**Figure 1:**
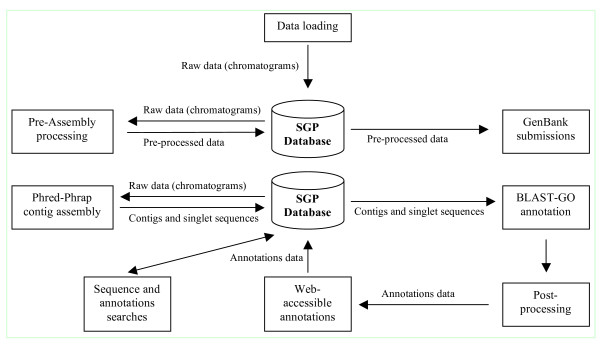
**SGP data flow**. The SGP data resource includes sequence processing and annotation pipelines, project and publicly available tools, and the project database.

The SGP data resource main components are the project database and data delivery system, large-scale sequence processing, clustering and annotation pipelines, and the web-based software tools [10]. The resource data flow is shown in Figure 1. All trace files or chromatograms (raw data) were loaded into the SGP database using the web-based submission interface. After loading the chromatograms were pre-processed using the SGP pre-processing pipeline preAssemble [11] which utilises Staden Pregap4 [16,17]. The pipeline was developed for automatic large scale processing of the DNA trace files and produces detailed output, which can be viewed with a web browser. PreAssemble also outputs sequences in a format ready for submission to GenBank. PreAssemble is available as a public web-based service and can also be downloaded as a stand-alone version as shown in Table 2. Pre-processing includes base calling and quality (confidence values) assignment by Phred [18,19], sequence quality clipping, sequencing vector clipping and poly A (T) tail clipping. Quality clip operates by first identifying the highest quality point in a sequence and moving a sliding window of 50 bases in both directions from this region, until the average quality for the window is below the minimum average confidence value. The minimum average confidence value of 15 was used. The quality clipped 5' and 3' ends of the sequence are marked. Vector clip uses the vector-primer data to identify and mark sequencing vector at both ends of the sequence. The remaining "good" insert sequence is again scanned for vector and if found, the sequence is rejected as failed. In the majority of cases this signifies that the sequence is mostly vector. Poly A (T) tail clip marks the consecutive occurrence of A of length 10 and above. If after these steps the remaining sequence length was less than a minimum value (preAssemble default 100 bases) it was also rejected as "failed". The pre-processed sequences that passed this screening scheme were submitted to GenBank after excluding ribosomal and mitochondrial sequences.

**Table 2:** Access to the Salmon Genome Project web resources.

**Salmon Genome Project (SGP) data resource**	
**Option name and description**	**Access from menu on the main page**

**SGP data and results statistics**	Data and results > Results > General statistics; Sequence length statistics Data and results > cDNA libraries > SGP libraries list short; SGP libraries list full

**Database search** SGP database text queries on sequence descriptions and annotations best hits, or searches of the SGP and NCBI sequence databases (Sequence search)	Database search > Simple search; Advanced search; Sequence search

**ESTs submitted to GenBank**	Data and results > Results > EST sequences in GenBank; Sequences in UniGene

**cDNA libraries** Description and number of ESTs in SGP libraries, pre-processed sequences, clustered and singlet sequences, sequences submitted to GenBank	Data and results > cDNA libraries > SGP libraries list short; SGP libraries list full > select library Data and results > cDNA libraries > SGP libraries > select library

**Clustered data datasets** Description and access to database snapshots of clustering and automatic annotation results for specific sets of data	Data and results > Clustered data datasets

**Clustered data summary** Clustering data and annotation best hits results for datasets Contigs and singlets annotation Contigs length and number of reads	Data and results > Clustered data summary > SGP > > Contigs and best annotation hits; Singlets and best annotation hits > Contigs length and number of reads; Distribution of average length and number of reads in contigs

**Contig and singlet sequences** Singlets and contig consensus sequences resulting from contig assembly of data from SGP cDNA libraries	Data and results > Clustered data datasets > Contigs; Singlets Data and results > cDNA libraries > SGP libraries (or select library) > Clustered and singlets sequences SGP Database > Search SGP database > Advanced search

**Annotations** Detailed BLAST-GO automatic annotation results and annotation statistics for datasets	Data and results > Annotations Database search > Simple search; Advanced search

**preAssemble** Pre-assembly automatic sequencer data processing pipeline available as a web-based tool and for downloading	SGP workbench > preAssemble Supplemental > Download preAssemble

**SGP local BLAST server** BLAST queries against the SGP local database, *Salmonidae*-specific and other NCBI datasets	SGP workbench > Blast search

Clustering and contig assembly of the screened sequences were performed with Phred-Phrap [20] packages. The repeat stringency 0.95 and minmatch 50 parameters were used for the Phrap contig assembly. The resulting contigs and singlet sequences were loaded into the SGP database as the SGP dataset after visual inspection and editing. The contigs and singlets were then submitted to the BLAST-GO annotation pipeline. Before loading into the database, singlet sequences were replaced by the matching pre-processed sequences from the database. Low quality ends were removed from the consensus contig sequences.

### Automatic annotation and datasets

The SGP high-throughput annotation pipeline operates by running local BLAST [21] searches against the NCBI PDB, SWISS-PROT, NR protein sequence and NR nucleotide sequence databases (NT). A stringent E-value threshold of 10^-15 ^was used as a criterion of significant matches for all databases except PDB where it was relaxed to 10^-10 ^in a bid to identify more of valuable matches with a known three-dimensional structure. These threshold values correspond to a range of very reliable homology matches [22]. The results were post-processed to show matches in the following order of potential importance and annotation value: PDB – SWISS-PROT – NR – NT. To identify biologically relevant matches, length of the segment of a query sequence aligned with the matching target in the database was calculated as per cent of the query length as well as the alignment length in bases. These results formed the basis for putative functional annotations by association. For the matches found in SWISS-PROT (a subset of UNIPROT) the Gene Ontology (GO) [23] definitions and relationships were extracted from the GO dataset if a GOA [24,25] assignment was present for the matching UNIPROT sequence (GO hits). Putative assignments were made from the GO subsets [GO: Molecular function], [GO: Biological process] and [GO: Cellular component]. Results were then processed by the annotation pipeline into web-based tables and loaded into the SGP database.

Sequence and annotations data are stored as "datasets" in the SGP data resource. Each dataset represents a set of data used for clustering and/or annotation. Datasets can overlap, e.g. a dataset selected for clustering can be formed from a subset of another larger clustered dataset or several datasets. If a specific new annotation is performed on a previously clustered and annotated dataset, it will form a new dataset with the same clustering results but different annotation. Such data design allows for consistently storing, accessing and comparing results data, which have fully or partially identical sets of initial trace data. All results data is accessible on the SGP web site; the detailed description of how to access various types of data is given in Table 2.

## Utility and discussion

### cDNA libraries and sequencing

From 23 libraries constructed from 15 different tissues we picked and stored approximately 155,000 clones in 384 well microtiterplates. Nineteen libraries were constructed from normal tissues and 4 libraries were constructed from gills and intestine using Suppression Subtractive Hybridisation (SSH) after intra-peritoneal injection of infectious salmon anaemia virus (ISAV) [15]. All libraries were constructed using fish from the pre-smolt stage. In addition, one library from each of the 15 tissues was also amplified in order to screen these for other genes in the future. From each library 50 tubes containing 1 ml each were stored at -80°C for use by anyone interested in this resource.

We performed 5' sequencing for approximately 75,000 pre-smolt cDNA clones from these libraries. Approximately 68,500 sequences (raw data) were loaded into the Salmon Genome Project (SGP) database as described in Construction and content. After loading, all sequences were subjected to pre-processing in order to clean out poor quality sequences and to trim off vector and linker sequences. After pre-processing there remained 58,109 (84%) high quality sequences, which have been marked as pre-processed ("passed") in the SGP database. The pre-processed sequences were submitted to the GenBank dbEST [26] after removal of the mitochondrial sequences (55,118 ESTs). The accession numbers are [GenBank: CK873405–CK900547, CN181049–CN181464, CO469580–CO472623, DN138771–DN140628, DN162055–DN167138, DN550291–DN550634, DV105941–DV107615, DW005391–DW007928, DW338860–DW340703, DW177413–DW183431, DW468731–DW473883]. Table 1 lists the total number of sequences, number of failed sequences, number of sequences submitted to GenBank and number of sequences used in the clustering from each of the 23 libraries.

### Clustering and contig assembly

**Figure 2:**
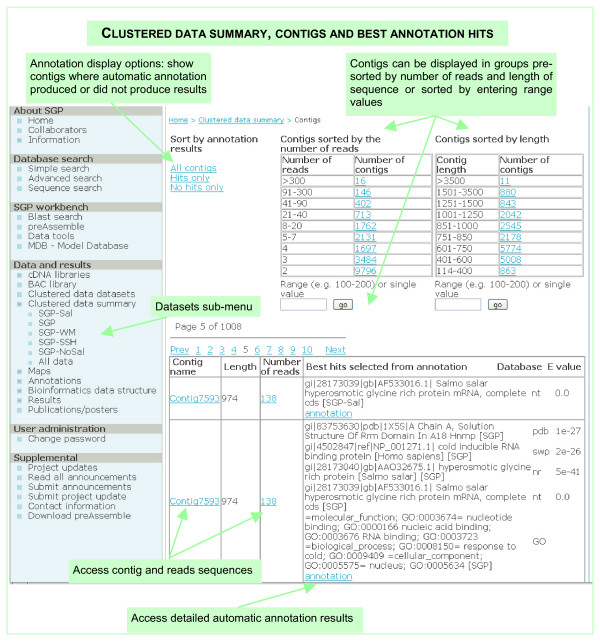
**Clustered data summary menu**. "Contigs and best annotation hits" display for the SGP dataset. Similar results display – "Singlets and best annotation hits" is available for singlets. Other options are "Contigs length and number of reads" and "Distribution of average length and number of reads in contigs". The Clustered data summary provides a current snapshot of the SGP database.

The project relies heavily on bioinformatics data processing and analysis and we have constructed the SGP data resource (Construction and content), which was used for the sequence processing, contig assembly, annotation, and project data hosting. All sequences, as well as other data and results, can be accessed through the SGP data resource as described in Table 2. Links in most of the web-based displays and tables show the results of online queries on the SGP database executed every time the table or link is accessed, i.e. they represent current "snapshots" of the SGP database. The SGP database and web site are updated on a regular basis with new data. Clustering and contig assembly was performed on 57,212 EST sequences from the 23 libraries shown in Table 1. The dataset contained raw sequences, for which the pre-processed sequences passed the preAssemble [11] processing criteria of sequence quality and vector contamination (Construction and content). Contig assembly resulted in 20,119 unique sequences, consisting of 6,424 contigs (32%) and 13,595 singlets (68%). This is a somewhat lower ratio of contigs compared to the GRASP results for Atlantic salmon [9] and our previous results of clustering of the combined GRASP and SGP ESTs [14]. The number of reads in each contig ranged from 2 to 508 with 49% of the contigs containing only 2 reads, 40% contained from 3 to 9 reads and 11% contained more than 10 reads. The length of the contigs varied from 3862 (4 contigs) to 103 (3 contigs) nucleotides with 67% of the contigs comprising between 600 and 1,500 nucleotides. A table listing number of contigs and average length for each observed number of reads per contig can be accessed as shown in Table 2, Clustered data summary. Contigs pre-sorted by length and number of reads as well as an option to sort into user defined intervals are available in the Clustered data summary menu, Contigs and best annotation hits, Figure 2.

### Annotation

**Figure 3:**
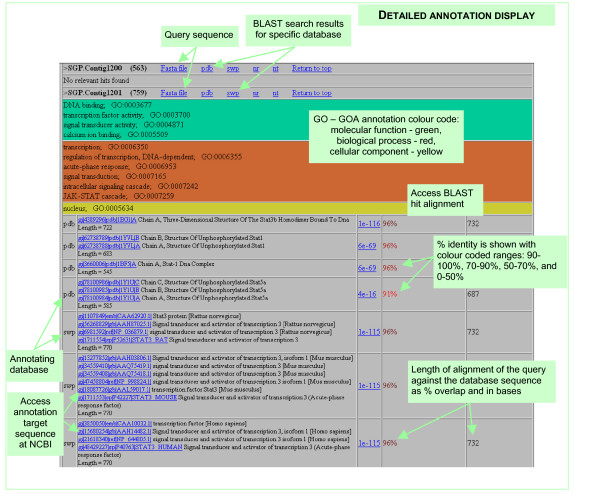
**Detailed automatic annotations display shows results of the GO – GOA and NCBI BLAST annotations**. Query and target sequences and complete BLAST results can be accessed from this page. Access to the detailed automatic annotations display for the full SGP annotation is available from SGP data resource > Data and results > Annotations > SGP full annotation. Links to the detailed annotation for the specific contig and singlet sequences are provided in the Annotation best hits display and for sequences accessed from the Clustered data datasets display and SGP database search results. Explanation of the format including BLAST parameters, table columns and colour coding is given at the top of each annotation page.

Contig consensus sequences and singlet sequences were annotated by the BLAST-GO automatic annotation pipeline. The annotation focused on the specialised, detailed results, which provide novel information and extend the currently available annotations data for *salmonidae*. The matches in PDB and SWISS-PROT databases were considered to be potentially more informative. A match in the PDB database leads to the PDB entry, which contains a link to the UNIPROT entry and, depending on the length of aligned query sequence opens a possibility of further function prediction by protein structure modelling. A SWISS-PROT match allowed access to detailed annotation data for the match sequence verified to high quality standards, including function assignment and cross-links with other databases. Where sequences in SWISS-PROT had GO terms [23] assigned by the GOA project [24,25], a putative function prediction of gene products was made and relevant GO terms were linked to the query sequence. The resulting annotation "best hits" and GO assignments were loaded into the SGP database. Database snapshots of the annotation data for datasets are accessible via the Clustered data datasets and Clustered data summary menus. See Table 2 for explanations on how to access all data and results. Searches of the SGP data can be done on specific annotation terms using the Database search option. The detailed annotation results are also accessible in the form of self-explanatory web-based tables in the Annotation section (Figure 3). GO-GOA assignment tables are available as part of the annotations. A *salmonidae*-specific search with the SGP dataset was performed on the NCBI NT database as an attempt to identify the SGP sequences which are similar to those that had previously been annotated as belonging to *salmonidae*. The results of this automatic annotation, loaded in the SGP database as the SGP-Sal dataset inevitably include some mismatches in its 1,768 hits, but provide a useful estimate of the possibly known *salmonidae *genes in the SGP data. Another annotation was performed where the sequences with *salmonidae *hits were excluded from the SGP dataset. The dataset identified as SGP-noSal was also loaded. These annotation datasets are available under the Clustered data datasets, Clustered data summary and Annotations menus, as well as via searches in the SGP database.

**Figure 4:**
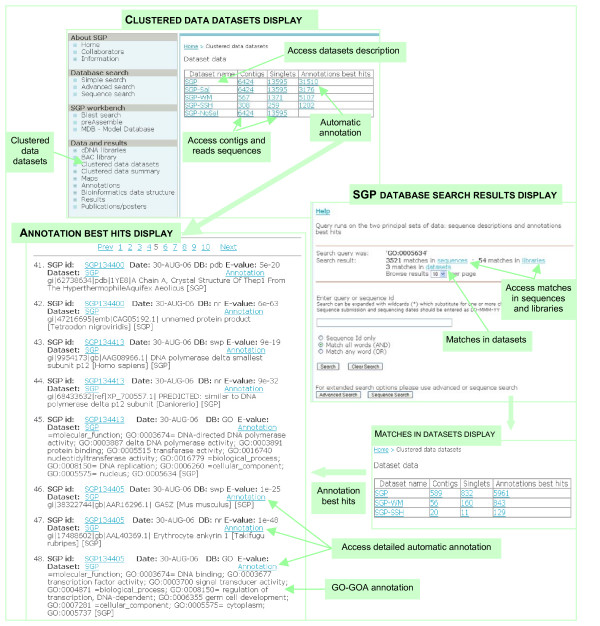
**Clustered data datasets menu gives access to dataset descriptions, and full sets of contigs and singlets sequences and automatic annotation results for each dataset**. Searches in the SGP database run on the three linked categories of data: sequences including their descriptions, libraries and annotations. When a match occurs in any of these data, all three data categories are shown in results. The "Matches in datasets" display provides access to a subset of contigs, singlets and annotations selected in the search. The "Annotations best hits" display is the same for the Clustered data datasets menu and database search results. Clustered data datasets menu provides a current snapshot of the SGP database.

There are three major ways of accessing the SGP data resource – by the Clustered data datasets and Clustered data summary menus, and via the SGP database searches. The Clustered data datasets menu (Figure 4) provides a database display with access to the contigs, singlets and annotations data for the datasets SGP, SGP-Sal and SGP-noSal described in this paper as well as other datasets. The Clustered data summary menu offers a different view on the data for each dataset with separate displays for dataset snapshots of the contigs and singlets best annotation hits, and distribution of the contigs length and number of reads (Figure 2). The displays allow interactive sorting of the data, and provide links both to the source sequence data and detailed annotations data. The SGP database searches are a flexible tool for accessing specific sequence or annotation data. Queries run on the two principal sets of data: sequence descriptions and annotations best hits, including GO-GOA annotations. Help on searching the database is available on the query interface web page.

The detailed annotation output format includes length of the matched segment of the query and per cent identity with the aligned sequence (Figure 3). Therefore a tentative selection of sequences for protein structure modelling can be made directly on the basis of annotation data. Since all SWISS-PROT sequences for which a similarity model can be built are modelled by the SWISS-MODEL automatic server [27] with results available from the SWISS MODEL Repository [28], a fairly good estimate of the quality and biological significance of modelling for these sequences can be made. As an example of such annotation, most of the larger SGP contigs (Clustered data summary > SGP > Contigs and best annotation hits) display hits in PDB and are suitable for modelling of at least part of their sequences.

Annotation statistics are shown in Table 3. On the whole for 50.3% sequences significant similarity was found in annotation databases. Since strict similarity threshold criteria were used in the BLAST annotation (Construction and content) this result although highly reliable, will tend to under-represent the number of possible annotated sequences. The contigs and singlets, which remain not annotated, potentially but not necessarily point to new genes. The rate of all annotation hits in contigs is predictably higher at 68%, with 42% in singlet sequences. Looking at highly informative hits in PDB or SWISS-PROT, more than 30% of all sequences returned hits in at least one of these databases, with contigs having a much higher rate of hits compared to the singlets. Of these hits 30% for contigs and 10% for singlets were in the PDB, allowing potential protein structure modelling or extraction of existing models from models databases such as SWISS MODEL Repository and ModBase [29].

**Table 3:** SGP automatic annotation statistics.

Database	% Annotated (automatic annotation)		
	Contigs and singlets	Contigs	Singlets

GO-GOA	30.5	48.5	22.0
pdb or swiss-prot	32.6	51.1	23.9
pdb	17.3	31.4	10.7
swiss-prot	31.7	50.1	23
nr	41.1	60.3	32.1
nt	36.7	53.7	28.6
Distribution between databases			
pdb	17.3	31.4	10.7
swiss-prot	15.3	19.7	13.2
nr	8.9	9.6	8.6
nt	8.8	7.7	9.3
any database (pdb + swiss-prot + nr + nt)	50.3	68.4	41.8
no hits	49.7	31.6	58.2

Detailed SGP dataset annotation statistics is available at SGP data resource > Data and results > Annotations > SGP full annotation, statistics.

**Databases**. NCBI databases – **pdb**: RCSB-PDB; **swiss-prot**: SWISS-PROT protein sequence database; **nr**: all non-redundant GenBank CDS translations + RefSeq Proteins + PDB + SwissProt + PIR + PRF; **nt **(nucleotide sequences): all GenBank + RefSeq Nucleotides + EMBL + DDBJ + PDB sequences (excluding HTGS0,1,2, EST, GSS, STS, PAT, WGS), no longer "non-redundant". **GO-GOA**: Gene Ontology assignments for the UNIPROT database produced by the GOA project.

**% Annotated**. Calculated as the number of sequences with successful annotations for a given subset (i.e. contigs and singlets, contigs, singlets) in a given database to the total number of sequences of this subset where annotation was attempted; GO annotation statistics was calculated separately from other databases as GO hits/GO no-hits. BLAST threshold E-values of 10^-10 ^for PDB and 10^-15 ^for other databases were used.

**Distribution between databases**. Only one successful annotation per sequence was counted, in the following ranking order: pdb OR swiss prot OR nr OR nt.

**SGP dataset**. 20019 contig consensus and singlet sequences.

**Figure 5:**
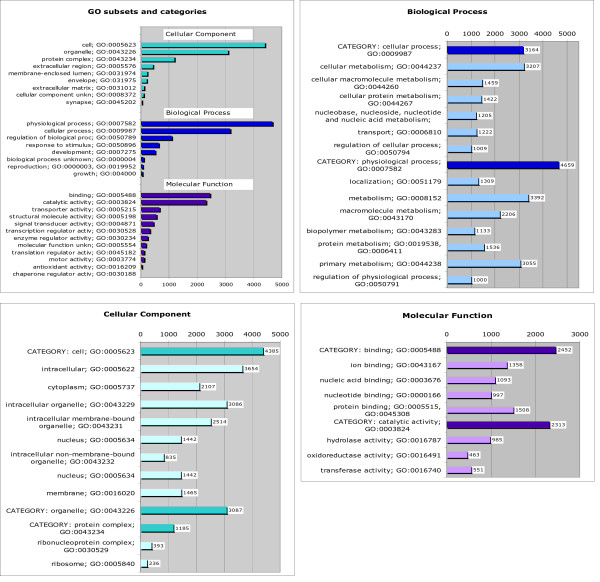
**GO-GOA automatic annotation results**. Upper level GO categories assignments, and the breakdown for each subset are shown for the three GO subsets, [GO: Molecular Function], [GO: Biological Process] and [GO: Cellular Component]. The annotation was performed for contig and singlet sequences. Complete SGP GO-GOA annotation is available at SGP data resource > Data and results > Annotations > SGP full annotation > GO term tables.

Putative assignments of the GO terms (Construction and content) referred to here as GO-GOA annotation were made for 30% of the SGP contigs and singlets, with almost 50% of the contigs returning GO-GOA hits. The GO-GOA annotations present a very uneven picture, Figure 5. Access to the detailed GO-GOA annotations is provided from the Annotation pages. The highest hit rates in the subset [GO: Molecular function] appear in the upper-level category [GO: binding] where they are distributed almost uniformly between child terms [GO: ion, nucleic acid binding] and [GO: protein binding], and another upper level category [GO: catalytic activity] in child terms [GO: hydrolase, oxidoreductase] and [GO: transferase activity]. Some of these peaks and GO terms frequencies e.g. binding and protein binding are in agreement with the GRASP GO Molecular function annotation, whereas other show markedly different patterns [9]. Hits in the categories [GO: structural molecule activity] and [GO: transporter activity] were relatively less frequent. The most under-represented molecular function categories in our current dataset are the [GO: chaperone regulator activity] and [GO: antioxidant activity]. In the GO subset [GO: Biological process] two of the upper-level categories are abundantly represented. In the [GO: cellular process] category the highest hit rates occurred in the child terms [GO: cellular metabolism, cellular macromolecular metabolism] and [GO: cellular protein metabolism]. Terms [GO: transport] and [GO: regulation of cellular physiological process] also occur frequently. The [GO: physiological process] category is again dominated by hits in the term [GO: cellular physiological process] and its lower-level child terms [GO: cellular metabolism, cellular macromolecule metabolism, cellular protein metabolism]. These terms are not shown in Figure 5 since they have multiple parents and also appear in category [GO: cellular process]. Another highly populated sub-category is [GO: localization]. Comparison of the GO numbers associated with similar terms in categories [GO: cellular process] and [GO: physiological process] such as [GO: metabolism, protein metabolism] suggests that these function assignments mostly concentrate in the cell. Term [GO: macromolecule metabolism] on the contrary shows relatively high occurrence outside of the [GO: cellular process]. The potentially important categories which are under-represented in the dataset are [GO: growth] and [GO: reproduction]. In the subset [GO: Cellular components] (indicating cellular localisation) most hits belong to the category [GO: cell], branching into child items [GO: intracellular, cytoplasm, intracellular organelle, intracellular membrane-bound organelle, nucleus], and category [GO: organelle] with the same child terms. Upper-level categories [GO: extracellular region] and [GO: extracellular matrix] have low hit rates compared to other categories.

Important function categories such as development, immune response and other were selected to assess the difference between annotations searches with the keywords representing broader search terms and alternatively exact GO terms. Results, listed in Table 4 show that as expected the broader search terms return for some queries substantially higher numbers of hits, which although not free from mismatches allow to identify most of the potentially relevant sequences. A search on GO terms returns only sequences with these exact GO terms in their annotations, allowing precise identification of particular GO assignments. The results display a clear division between the frequencies of putative function assignments in the SGP data. Highly represented hits for more general functions such as development, growth, differentiation, proliferation, predictably stand out in comparison with the lower numbers for more narrow categories e.g. response to stress, inflammatory response, cell growth. Some very low hit numbers for the exact GO terms are markedly different from similar broader terms, such as immune cell activation, growth, regulation of growth, regulation of cell differentiation. An important result is the high number of hits for a relatively narrow GO term immune response, i.e. higher than average representation of this function in the SGP dataset. The SGP ESTs thus can be used as a source for mining immune-related genes. The keyword "sex" returned few hits both for general and exact GO searches, suggesting that the relevant functions are under-represented in this dataset.

**Table 4:** Annotations search results for a representative sample of potentially important functions.

**Search term**	**Contigs**	**Singlets**	**Contigs and singlets**	**GO definitions (top category GO:0008150 : biological process)**
immune response	94	64	153	
immune response GO:0006955	91	60	147	GO:0007582 : physiological process GO:0050874 : organismal physiological process GO:0002376 : immune system process GO:0006955 : immune response GO:0051869 : physiological response to stimulus GO:0006955 : immune response
immune cell activation GO:0045321	0	1	1	GO:0007582 : physiological process GO:0050874 : organismal physiological process GO:0001775 : cell activation GO:0045321 : leukocyte activation GO:0002376 : immune system process GO:0045321 : leukocyte activation (Exact synonym: immune cell activation)
response to stress	28	28	56	
response to stress GO:0006950	14	13	27	GO:0050896 : response to stimulus GO:0006950 : response to stress
inflammatory response	29	24	53	
inflammatory response GO:0006954	26	21	47	GO:0007582 : physiological process GO:0050874 : organismal physiological process GO:0006954 : inflammatory response
response to virus	7	8	15	
response to virus GO:0009615	7	5	12	GO:0050896 : response to stimulus GO:0009607 : response to biotic stimulus GO:0051707 : response to other organism GO:0009615 : response to virus
wounding	2	3	5	
response to wounding GO:0009611	2	3	5	GO:0050896 : response to stimulus GO:0009605 : response to external stimulus GO:0009611 : response to wounding
development	139	192	331	
development GO:0007275	73	120	193	GO:0007275 : development
growth	74	128	202	
growth GO:0040007	1	0	1	GO:0040007 : growth
cell growth	32	41	73	
cell growth GO:0016049	6	1	7	GO:0040007 : growth GO:0016049 : cell growth
regulation of growth	26	41	67	
regulation of growth GO:0040008	0	2	2	GO:0040007 : growth GO:0040008 : regulation of growth GO:0050789 : regulation of biological process GO:0040008 : regulation of growth
differentiation	71	90	161	
regulation of differentiation	35	48	83	
cell differentiation GO:0030154	41	52	93	GO:0009987 : cellular process GO:0030154 : cell differentiation GO:0007275 : development GO:0030154 : cell differentiation
regulation of cell differentiation GO:0045595	0	2	2	GO:0009987 : cellular process GO:0030154 : cell differentiation GO:0045595 : regulation of cell differentiation
proliferation	86	87	173	
regulation of proliferation	61	65	126	
cell proliferation GO:0008283	37	37	74	GO:0009987 : cellular process GO:0050875 : cellular physiological process GO:0008283 : cell proliferation GO:0007582 : physiological process GO:0050875 : cellular physiological process GO:0008283 : cell proliferation
regulation of cell proliferation GO:0042127	5	4	9	GO:0007582 : physiological process GO:0050875 : cellular physiological process GO:0008283 : cell proliferation GO:0042127 : regulation of cell proliferation
sex	14	7	21	
sex determination GO:0007530	2	1	3	GO:0007275 : development GO:0007530 : sex determination

Searches were done for most of the queries on a wider term and on an exact GO term in the subset [GO: biological process]. Although wider search terms are bound to produce some mismatches, they can be more useful in identifying all important matches.

## Conclusion

We have constructed 23 tissue specific cDNA libraries from pre-smolt Atlantic salmon (*Salmo salar*). Subsequent EST sequencing and clustering yielded 6,424 contigs and 13,595 singlets, resulting in a total of 20,119 unique sequences.

Putative annotation was assigned to 50.3% of the sequences showing similarity to known genes, mostly from other species, in one or more of the databases used for automatic annotation. 30.5% of sequences were further annotated using annotation transfer procedure for Gene Ontology (GO) terms for molecular function, biological process and cellular component.

All data on ESTs, clustering and annotation can be accessed via the SGP data resource [10]. There is a variety of data access options such as database searches on annotation including gene assignments and GO terms as well as access to self explanatory web-based detailed annotation archives. Annotation searches on biologically important putative functions showed that the [GO: immune response] term is over-represented in the SGP dataset, suggesting the presence of a relatively high number of immune-related genes.

On the whole, annotation searches in the SGP database and access to annotations as datasets summary or as detailed results offer a powerful tool for exploring, at different levels of granularity, biological features reflected in the EST data. A database search, which can be done using sophisticated keywords search options, will produce an overview of the highly reliable sequence similarities ("best hits") and their gene and function annotations including GO assignments. For each of the "best hits" displayed in the overview, a separate link will produce a detailed annotation output presented in a user-friendly format, listing all significant hits in all databases used in the annotation. Users wishing to explore further annotation details can do this via links to the source EST sequences, dissected alignments in the original BLAST format, target (hit) sequences in the source databases, and the original GO annotation tree.

## Availability and requirements

The Salmon Genome Project (SGP) data resource is available at .

The web access is optimised for Netscape 8 and Internet Explorer.

## Authors' contributions

AAA supervised SGP bioinformatics, designed software and data processing techniques and developed the data resource. He carried out software development and data processing and drafted part of the manuscript.

AVV, TAR, and JKL worked on software design, carried out development, implementation and data processing. AVV in addition were responsible for web design.

HH-L did the construction and sequencing of the SSH as well as the normal gills and intestine libraries.

BH conceived, headed and coordinated the project and performed the construction and sequencing of all cDNA libraries except those done by HH-L. He loaded all sequences into the DB and performed the sequence processing using the preAssemble pipeline. He drafted part of the manuscript.

All authors have read and approved the final manuscript.
